# Survival of SARS-CoV-2 on Clothing Materials

**DOI:** 10.1155/2021/6623409

**Published:** 2021-04-08

**Authors:** Jenni Virtanen, Kirsi Aaltonen, Ilkka Kivistö, Tarja Sironen

**Affiliations:** ^1^Department of Veterinary Biosciences, Faculty of Veterinary Medicine, University of Helsinki, Agnes Sjöbergin Katu 2, Helsinki 00790, Finland; ^2^Department of Virology, Faculty of Medicine, University of Helsinki, Haartmaninkatu 3, Helsinki 00290, Finland

## Abstract

In order to plan and execute proper preventative measures against COVID-19, we need to understand how SARS-CoV-2 is transmitted. It has been shown to remain infectious on surfaces from hours to days depending on surface type and environmental factors. The possibility of transmission through fur animals and contaminated pelts, along with the safety of those working with them, is a major concern. SARS-CoV-2 can infect minks and raccoon dogs and has spread to mink farms in numerous countries. Here, we studied the stability of SARS-CoV-2 on blue fox, Finn raccoon, and American mink pelt, fake fur, cotton, plastic, faux leather, and polyester and tested its inactivation by UV light and heat treatment. We detected infectious virus up to 5 days on plastic, up to 1 day on fake fur, less than a day on cotton, polyester, and faux leather, and even 10 days on mink fur. UV light failed to inactivate SARS-CoV-2 on pelts, most likely due to the mechanical protection by the fur. Hence, it should not be used to inactivate the virus on fur products, and its use for other surfaces should also be considered carefully. Heat treatment at 60°C for 1 h inactivated the virus on all surfaces and is a promising method to be applied in practice. This study helps prevent further spread of COVID-19 by increasing our understanding about risks of SARS-CoV-2 spread through contaminated clothing materials and giving important information needed to improve safety of those working in the production line as well as people using the products.

## 1. Introduction

Better understanding about the transmission of severe acute respiratory syndrome coronavirus 2 (SARS-CoV-2) is needed to be able to set up proper preventative measures. The role of surface contamination has been discussed as several studies have found SARS-CoV-2 genetic material on various surfaces, though most of those studies have focused on genetic material and not the infectious virus [1–3]. Based on studies about SARS-CoV-2 infectivity on surfaces performed in laboratory settings, stability on surfaces can vary from hours to days. It has stayed infectious for several days on plastic, steel, and outer layer of surgical mask, but only from some hours to a couple of days on paper, wood, and cloth [4–6]. Environmental conditions like temperature and humidity have also been shown to affect SARS-CoV-2 infectivity, and the virus is inactivated faster in higher temperature and humidity [4, 5]. In studies performed with other coronaviruses, results have also varied depending on the surface, environmental conditions, and the virus. On plastic, Middle East respiratory syndrome coronavirus (MERS-CoV) and HCoV-229E lost their infectivity within 72 hours, whereas severe acute respiratory syndrome coronavirus (SARS-CoV-1) stayed viable from three days to over two weeks [6–10].

The possibility of transmission of SARS-CoV-2 through fur animals, contaminated pelts, and fur products as well as the safety of the people working with them has been a cause of concern as SARS-CoV-2 infects mink and raccoon dogs. The disease has spread widely in mink farms in the Netherlands and Denmark and has also been reported in Spain, France, Italy, Sweden, Greece, Canada, Lithuania, and Poland. Evidence of both human-to-mink and mink-to-human transmission has been reported [11–14].

Our aim was to study SARS-CoV-2 stability on fur and a set of different fabrics and its inactivation by UV light and heat treatment to gain information about SARS-CoV-2 survival and possible ways to inactivate the virus. It is important to understand SARS-CoV-2 stability and inactivation on clothing materials to be able to assess risks of virus transmission through them during the production line and clothing stores where several people touch the same products. Information about SARS-CoV-2 stability on different fabrics is also needed to define suitable materials for reusable masks.

## 2. Materials and Methods

### 2.1. Cell Line, Virus Stock, and Surface Materials

Vero E6 cells were grown in minimal essential eagle's medium (MEM, Sigma-Aldrich) with 10% fetal bovine serum (FBS, Gibco), penicillin, and streptomycin. Cells were infected with SARS-CoV-2/Finland/1/2020 isolate [15] (passage 7), grown for three days in MEM with 2% FBS and antibiotics, collected when around 90% of cells were detached, and centrifuged at 3220 g for 15 min. Supernatant was used as a virus stock and titer was determined to be 10^6^ PFU/ml by end-point titration. The pelts of American mink (*Neovison vison*), blue fox (*Vulpes lagopus*), and Finn raccoon (*Nyctereutes procyonoides*) were obtained directly from the Finnish Fur Breeders' Association and the rest of the fabrics were obtained from a fabric store. Approximately 5 cm^2^ pieces were cut for the experiment. All the virus culturing was performed in a BSL3 laboratory.

### 2.2. Virus Stability on Surfaces

Six 50 *µ*l drops of virus stock (one for each time point) were spiked and spread onto both sides of 5 cm^2^ pieces of American mink pelt, blue fox pelt, Finn raccoon pelt, and fake fur and on plastic Petri dishes. Pelts and fake fur were trimmed before the study to gain a better access to the surface. Virus suspensions were air-dried at room temperature (RT, temperature 21°C). One piece of each surface type did not get any treatment, one was held under UV-C lamp for 5 min, and one was incubated in 60°C water bath right above water level (Julabo TW12) for 1 h (temperature above water level around 51-52°C, humidity 99%). Samples were taken at 6 different time points after treatment (5 min, 30 min, 1 day, 3 days, 7 days, and 14 days) with sterile cotton swabs that were put in 300 *µ*l of the cell culture media and vortexed for a few seconds. Vero E6 cells were infected with 100 *µ*l of each sample in two parallel wells for 1 h at 37°C after which virus solutions were replaced with 100 *µ*l of MEM with 2% FBS and antibiotics. Virus stock with 1 : 500 dilution was used as a positive control and media as a negative control. Pelts, fake fur, and Petri dishes were kept at RT in a closed container and out of direct light between time points.

Study was repeated with both sides of American mink pelt, both sides of fake fur, faux leather, cotton, polyester, and surface of the Petri dish, and the protocol was modified by adding time points, measuring humidity and using dry heat for heat treatment. Time points included one time point each day for the first seven days and one after 10 days, humidity of the room fluctuated between 40 and 75% during the experiment, and heat treatment was carried out in a hybridization oven (Robbins Scientific, Model 1000) at 60°C for 1 h. UV treatment was not included, and mink pelts and fake fur were not trimmed.

Cells were incubated at 37°C for four days after which the plates were checked for cytopathic effect (CPE) with a microscope. To visualize the results, the plates were stained with crystal violet (Figure S1 in Supplementary Material) by incubating with 100 *µ*l of 36.5% formalin per well for 30 min and washing with 100 *µ*l of water followed by incubation with 100 *µ*l of 1 : 10 diluted crystal violet solution for 10 min and washing twice with 100 *µ*l of water.

All growth media of the first experiment and growth media from untreated samples of time points 0, 1, 2, 5, 6, 7, and 10 days of the second experiment were tested with PCR to make sure CPE was caused by SARS-CoV-2. RNA was extracted with QIAcube HT (Qiagen) using the QIAamp 96 Virus QIAcube HT kit (Qiagen) according to kit protocol. Lysis was performed off-board by adding 160 *µ*l of ACL buffer, 20 *µ*l of proteinase K, and 100 *µ*l of PBS into each sample with approximate volume of 100 *µ*l and incubating at RT for 30 min. RNAs were tested with SARS-CoV-2 N gene targeting PCR described by Corman et al. to confirm if CPE was caused by SARS-CoV-2 infection or some cytotoxic substance on the surface [16].

## 3. Results

Results of the first test on SARS-CoV-2 stability on surfaces without inactivating treatment and after treatment with heat or UV light are presented in Tables 1, S1, and S2. They were considered positive if CPE was detected, and Ct values of RT-PCR performed from growth media were less than 20.00 in one or both parallel reactions. If Ct value was over 30.00, the result was considered negative, as the RT-PCR signal likely resulted from original virus stock instead of an infectious virus. Positive results were detected after three days on fur side of Finn raccoon, blue fox, and mink pelt and on Petri dish. None of the samples were positive after one week. After UV treatment, CPE was detected in samples from fur side of Finn raccoon, blue fox, and mink pelt and fake fur, and after heat treatment, CPE was detected in mink pelt sample. Samples taken from skin side did not give any positive results after the first day.

In the second experiment (Table 2), samples taken from Petri dish without heat treatment were positive after 5 days, fur side of mink pelt after 10 days, fabric side of fake fur after 1 day, and skin side of mink pelt, fur side of fake fur, faux leather, and polyester only in the first day. None of the samples taken from cotton or after heat treatment were positive.

## 4. Discussion

To our knowledge, this is the first study on SARS-CoV-2 ability to survive on furs and compares a larger set of different clothing materials than previous studies. These results are important for international trade and work safety of the persons handling the pelts in the production end and give more information to evaluate risks of SARS-CoV-2 transmission through contaminated clothes and other fabrics. Even though this setup does not describe the situation where the fur animal itself has been infected, which would require an experimental infection, it gives important information about the situation where the virus ends up onto the fur or fabric from the environment, like dust in the air or humans working with the animals and their pelts.

Our result concerning plastic and cotton is consistent with previous studies making the results with pelts and other materials that have not been studied before also comparable. Similar to other studies, where SARS-CoV-2 survival time on plastic has varied between 3 and 7 days [4, 6], we found infectious virus from plastic for up to 5 days. We were unable to culture the virus from cotton even right after drying the virus onto the surface, which is also consistent with other studies stating that SARS-CoV-2 is more stable on smooth materials [4]. In addition to cotton, this was also observed with other absorbent materials like polyester, faux leather, and skin side of pelts from which infectious virus could not be cultured after the first day. Similar pattern about infectivity on cotton has earlier been detected with SARS-CoV-1 that, according to Lai et al., loses its infectivity faster on cotton gown than on disposable gown [17]. It is unclear whether the virus is inactivated or if it just does not detach from the fabric during the sampling, but if the virus is not caught by intense swapping, it is most likely not easily caught by, e.g., human fingers either. These results indicate that the risk of getting SARS-CoV-2 from contaminated cotton is small. It also supports the use of cotton, e.g., in reusable cloth masks, even though more thorough studies about different mask materials are needed. According to our results, risk of transmission through contaminated polyester or faux leather is also small compared to plastic but not nonexistent as infectious virus was detected in the first time point (after 30 min) and several people might be in contact with a material during that time.

Interestingly, the virus could be cultured from mink fur even after 10 days in the second experiment. Since we did not have any time points beyond that, some virus particles might have stayed infectious even longer. In the first experiment, infectious virus could not be found after 7 days making the time of infectivity between 3 and 6 days. The difference is probably due to the differences in experimental conditions like trimming the furs before treatment and fluctuating humidity in the room. Since checking the virus titers would not have been reliable in this setup due to the physical loss of virus into the fur or fabric, we do not know how fast the virus titer decreased during the experiment, but as CPE was always clearly detected after two or three days, the virus amount was sufficient to cause a strong infection in cells. It is not clear if the better stability on fur can be explained by, e.g., mechanical protection by the fur or the biological properties of the fur. As stability on fake fur was significantly decreased compared to the animal fur, biological properties and substances of the animal fur seem a more likely explanation than mechanical protection. On the skin side, however, the stability was not increased, but it might be that the virus was absorbed into skin and could not be swapped. Viral loads in real-life situations can be difficult to imitate as they vary a lot: viral loads in respiratory specimens have been reported to range from almost nothing to even 10^11^ copies/ml with PCR [18, 19]. This and the fact that the experimental setups and environmental conditions are not constant make it difficult to estimate the exact time of infectivity on different surface materials. However, the results between materials can be compared and it is evident that SARS-CoV-2 survives on mink pelt longer than other tested surfaces leading to the question if this stability contributes to the efficient spread of the virus within mink farms.

UV treatment inactivated SARS-CoV-2 on plastic but not on fur side of the pelts (Table 1), which is probably due to mechanical protection of the virus particle by the fur as similar lack of inactivation was also detected on fake fur. Based on this, UV treatment is not a suitable method to inactivate SARS-CoV-2 in fur or fake fur products and its use for other products should also be considered carefully if there is a risk that UV light cannot radiate the whole contaminated area.

Heat treatment in dry heat completely inactivated the virus on all materials, but inactivation on mink pelt in water bath was insufficient, which has several possible explanations. There was a big difference in humidity between water bath and hybridization oven, and temperature above water level was only around 51-52°C instead of 60°C. Based on earlier studies, increasing humidity accelerates coronavirus inactivation [5, 8], making a fluctuating temperature the more likely reason for insufficient inactivation of the virus in the water bath. The fur might also inhibit the heat from fully penetrating and the fat layer might protect the virus, as the virus was completely inactivated on plastic by both heat treatments. Our results show that heat treatment can be applied to inactivation of the virus on all tested materials, but it needs to be properly controlled to make sure that the inactivation is complete.

Handling and transporting some materials requires precaution due to the long survival time of SARS-CoV-2 on some surfaces to protect the persons handling the products and to prevent further spread of the virus. However, our study shows that there are ways to inactivate the virus that could also be implemented in practice. Further studies are needed to find out the reasons to varying preservation of infectivity of SARS-CoV-2 on surfaces, and several common materials still remain unstudied.

## 5. Conclusions

SARS-CoV-2 stability on tested clothing materials varied from minutes on cotton to days on pelts, and precautions need to be taken especially when handling fur animals and their pelts due to the long survival time of the virus. Risk of SARS-CoV-2 transmission through polyester, faux leather, and especially cotton is small due to the shorter time of infectivity.

Based on our results, UV-C light treatment is not suitable to inactivate the virus on fur due to the mechanical protection of the fur and its use for other than smooth surfaces should also be carefully considered. Properly controlled heat treatment, however, can be applied to protect the people handling fur animals and their products.

## Figures and Tables

**Table 1:** Stability of SARS-CoV-2 on surfaces without inactivating treatment and after inactivating treatments under UV light for 5 min and in 60°C water bath for 1 h.

Surface	Side	Inactivation	5 min	30 min	1 day	3 days	7 days	14 days
Finn raccoon pelt	Fur side	No inactivation	+	+	+	+	−	−
	Skin side		−	−	−	−	−	−
Blue fox pelt	Fur side		+	+	+	+	−	−
	Skin side		+	−	−	−	−	−
American mink pelt	Fur side		+	+	+	+	−	−
	Skin side		−	−	−	−	−	−
Fake fur	Fur side		+	+	−	−	−	−
	Skin side		+	+	−	−	−	−
Petri dish			+	+	+	+	−	−

Finn raccoon pelt	Fur side	UV treatment	+	+	+	−	−	−
	Skin side		+	−	−	−	−	−
Blue fox pelt	Fur side		+	+	+	+	−	−
	Skin side		−	−	−	−	−	−
American mink pelt	Fur side		+	+	+	−	−	−
	Skin side		−	−	−	−	−	−
Fake fur	Fur side		−	+	−	−	−	−
	Skin side		−	+	−	−	−	−
Petri dish			−	−	−	−	−	−

Finn raccoon pelt	Fur side	Heat treatment	−	−	−	−	−	−
	Skin side		−	−	−	−	−	−
Blue fox pelt	Fur side		−	−	−	−	−	−
	Skin side		−	−	−	−	−	−
American mink pelt	Fur side		+	+	+	−	−	−
	Skin side		−	−	−	−	−	−
Fake fur	Fur side		−	−	−	−	−	−
	Skin side		−	−	−	−	−	−
Petri dish			−	−	−	−	−	−

+: infectious virus detected; −: infectious virus not detected.

**Table 2:** Stability of SARS-CoV-2 on surfaces without inactivating treatment and after heat treatment at 60°C for 1 h.

Surface	Inactivation	0 days	1 day	2 days	3 days	4 days	5 days	6 days	7 days	10 days
Mink pelt, fur side	No inactivation	+	+	+	+	+	+	+	+	+
Mink pelt, skin side		+	−	−	−	−	−	−	−	−
Fake fur, fur side		+	−	−	−	−	−	−	−	−
Fake fur, fabric side		+	+	−	−	−	−	−	−	−
Faux leather		+	−	−	−	−	−	−	−	−
Cotton		−	−	−	−	−	−	−	−	−
Polyester		+	−	−	−	−	−	−	−	−
Petri dish		+	+	+	+	+	+	−	−	−

Mink pelt, fur side	Heat treatment	−	−	−	−	−	−	−	−	−
Mink pelt, skin side		−	−	−	−	−	−	−	−	−
Fake fur, fur side		−	−	−	−	−	−	−	−	−
Fake fur, fabric side		−	−	−	−	−	−	−	−	−
Faux leather		−	−	−	−	−	−	−	−	−
Cotton		−	−	−	−	−	−	−	−	−
Polyester		−	−	−	−	−	−	−	−	−
Petri dish		−	−	−	−	−	−	−	−	−

+: infectious virus detected; −: infectious virus not detected.

## Data Availability

The culturing data (parallel reactions combined) used to support the findings of this study are included within the article, and PCR results and the pictures of the crystal violet stained plates of the second experiment are given in the Supplementary Information file. Detailed culturing results (parallel reactions not combined) and the pictures of the plates from the first experiment are available from the corresponding author upon request.

## Supplementary Materials

Supplementary Materials Supplementary material containing pictures of the crystal violet stained plates and PCR results can be found online.

## References

[1] Chia P. Y., Coleman K. K., Tan Y. K. (2020). Detection of air and surface contamination by SARS-CoV-2 in hospital rooms of infected patients. *Nature Communication*.

[2] Guo Z.-D., Wang Z.-Y., Zhang S.-F. (2020). Aerosol and surface distribution of severe acute respiratory syndrome coronavirus 2 in hospital wards, Wuhan, China, 2020. *Emerging Infectious Diseases*.

[3] Ye G., Lin H., Chen S. (2020). Environmental contamination of SARS-CoV-2 in healthcare premises. *Journal of Infection*.

[4] Chin A. W. H., Chu J. T. S., Perera M. R. A. (2020). Stability of SARS-CoV-2 in different environmental conditions. *The Lancet Microbe*.

[5] Biryukov J., Boydston J. A., Dunning R. A. (2020). Increasing temperature and relative humidity accelerates inactivation of SARS-CoV-2 on surfaces. *mSphere*.

[6] Van Doremalen N., Bushmaker T., Morris D. H. (2020). Aerosol and surface stability of SARS-CoV-2 as compared with SARS-CoV-1. *New England Journal of Medicine*.

[7] Van Doremalen N., Bushmaker T., Munster V. J. (2013). Stability of Middle East respiratory syndrome coronavirus (MERS-CoV) under different environmental conditions. *Euro Surveillance*.

[8] Chan K. H., Peiris J. S., Lam S. Y., Poon L. L., Yuen K. Y., Seto W. H. (2011). The effects of temperature and relative humidity on the viability of the SARS coronavirus. *Advances in Virology*.

[9] Aboubakr H. A., Sharafeldin T. A., Goyal S. M. (2020). Stability of SARS-CoV-2 and other coronaviruses in the environment and on common touch surfaces and the influence of climatic conditions: a review. *Transboundary and Emerging Diseases*.

[10] Rabenau H. F., Cinatl J., Morgenstern B., Bauer G., Preiser W., Doerr H. W. (2005). Stability and inactivation of SARS coronavirus. *Medical Microbiology and Immunology*.

[11] (2021). Events in animals: OIE-world organisation for animal health. https://www.oie.int/en/scientific-expertise/specific-information-and-recommendations/questions-and-answers-on-2019novel-coronavirus/events-in-animals.

[12] Freuling C., Breithaupt A., Müller T. (2020). Raccoon dogs are susceptibleto and efficiently transmit SARS-CoV2 and may serve as intermediate host. *BioRxiv Preprint*.

[13] Oreshkova N., Molenaar R. J., Vreman S. (2020). SARS-CoV-2 infection in farmed minks, The Netherlands, April and May 2020. *Euro Surveillance*.

[14] Oude Munnink B., Sikkema R., Nieuwenhuijse D. (2020). Jumping back and forth: anthropozoonotic and zoonotic transmission of SARS-CoV-2 on mink farms. *BioRxiv*.

[15] Haveri A., Smura T., Kuivanen S. (2020). Serological and molecular findings during SARS-CoV-2 infection: the first case study in Finland, January to February 2020. *Euro Surveillance*.

[16] Corman V. M., Landt O., Kaiser M. (2020). Detection of 2019 novel coronavirus (2019-nCoV) by real-time RT-PCR. *Euro Surveillance*.

[17] Lai M. Y., Cheng P. K., Lim W. W. (2005). Survival of severe acute respiratory syndrome coronavirus. *Clinical Infectious Diseases*.

[18] Kleiboeker S., Cowden S., Grantham J. (2020). SARS-CoV-2 viral load assessment in respiratory samples. *Journal of Clinical Virology*.

[19] Pan B. X., Sadun A. A., Sebag J. (2020). Correspondence. *Retina (Philadelphia, Pa.)*.

