# Supplementary material for: Survival of SARS-CoV-2 on Clothing Materials
Source: Adv Virol. 2021 Apr 8;2021:6623409. doi: 10.1155/2021/6623409 (PMC8049815; doi:10.1155/2021/6623409)
Supplement: Supplementary Materials — Supplementary material containing pictures of the crystal violet stained plates and PCR results can be found online. [file 6623409.f1.docx]

# **Supplementary material: Survival of SARS-CoV-2 on clothing materials**


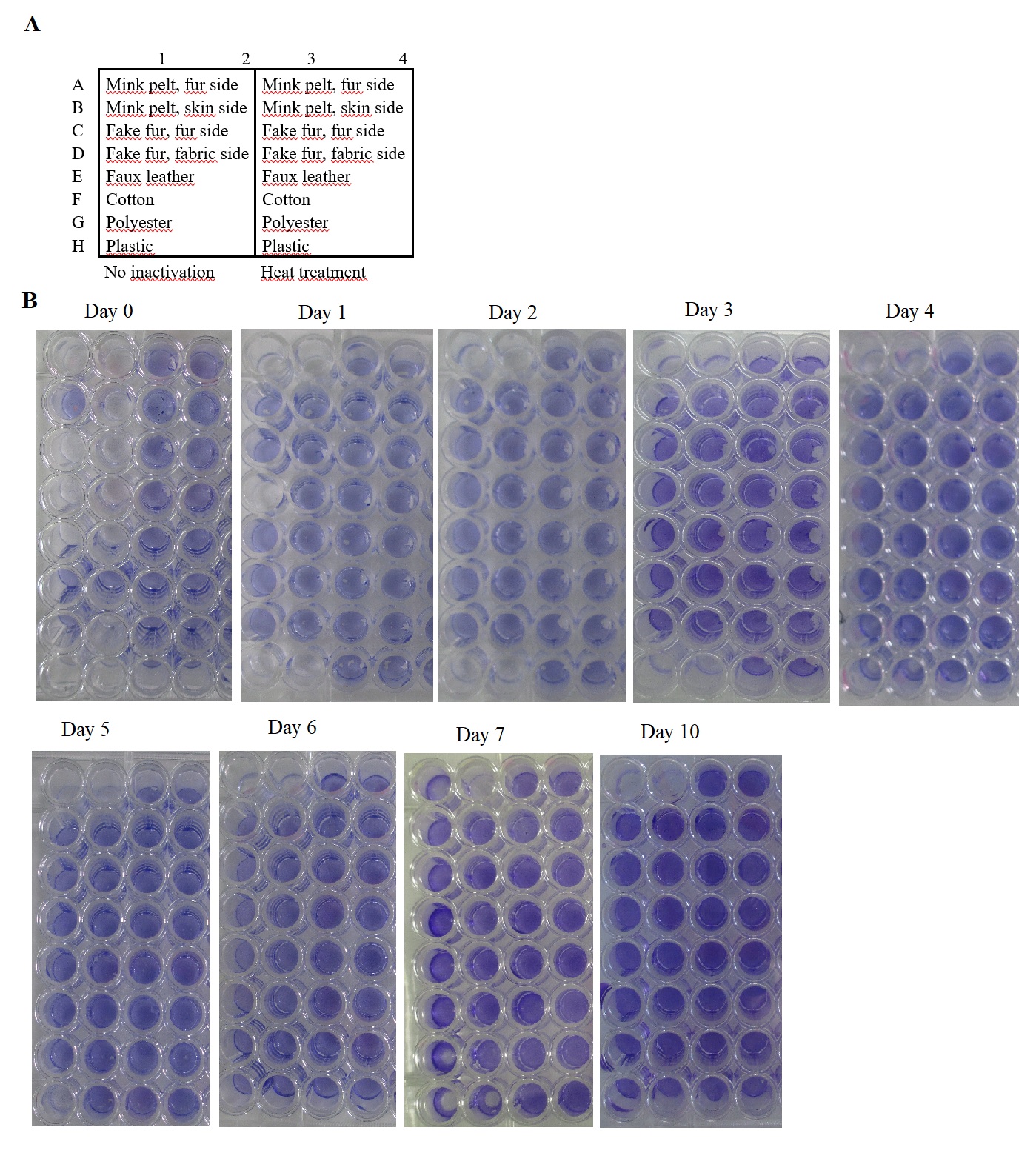


Figure S1: Sample order (A) and crystal violet stained virus cultures of each time point (B) of experiment 2. All the samples were analyzed in two parallel reactions. Blank color in the wells indicates the lack of cells due to the cytopathic effect caused by SARS-CoV-2, environmental contaminant, or mechanical disturbance.

Table S1: PCR results from the culture media of the test about the stability of SARS-CoV-2 on surfaces without inactivating treatment and after inactivating treatments under UV light for 5 min and in 60 ᵒC water bath for 1 h. Ct-threshold has been set to 0.02.

| Species | Surface | Treatment | 5 min | 30 min | 1 day | 3 days | 7 days | 14 days |
| --- | --- | --- | --- | --- | --- | --- | --- | --- |
| Finn raccoon pelt | Fur side | None | 17.77/17.38 | 17.76/16.78 | 16.91/16.55 | 16.21/15.90 | 31.73/31.00 | 32.75/34.88 |
|  | Skin side |  | 33.69/33.46 | 37.06/37.95 | no ct/36.07 | no ct | 38.88/38.04 | 38.78/35.98 |
|  | Fur side | UV | 17.73/17.93 | 17.06/17.07 | 23.13/16.34 | 32.60/32.79 | 31.30/30.40 | 33.55/34.71 |
|  | Skin side |  | 33.14/16.82 | 31.60/31.04 | no ct/no ct | 37.63/32.62 | no ct/no ct | 37.17/no ct |
|  | Fur side | Heat | 31.58/32.01 | 31.94/32.64 | 34.32/32.74 | 32.85/33.45 | 33.32/31.78 | 34.37/35.53 |
|  | Skin side |  | 38.37/38.49 | no ct/no ct | no ct/no ct | no ct/no ct | no ct/no ct | no ct/no ct |
| Bluefox pelt | Fur side | None | 17.23/17.38 | 17.33/16.67 | 15.28/16.25 | 16.20/17.54 | 33.18/33.90 | 33.32/34.08 |
|  | Skin side |  | 15.85/32.05 | 35.60/34.16 | no ct/no ct | no ct/36.50 | no ct/no ct | no ct/38.28 |
|  | Fur side | UV | 31.03/18.13 | 16.87/15.73 | 17.38/16.90 | 17.63/20.12 | 31.95/32.02 | 32.52/32.99 |
|  | Skin side |  | 33.42/33.59 | 35.46/35.79 | no ct/no ct | no ct/no ct | no ct/no ct | no ct/no ct |
|  | Fur side | Heat | 31.34/30.72 | 31.30/31.16 | 34.01/35.18 | 32.87/33.79 | 31.34/31.91 | 33.34/35.05 |
|  | Skin side |  | no ct/no ct | no ct/no ct | no ct/no ct | no ct/no ct | no ct/no ct | no ct/no ct |
| American mink pelt | Fur side | None | 17.08/16.50 | 17.56/15.93 | 16.63/21.45 | 34.75/16.16 | 31.60/30.40 | 34.24/37.16 |
|  | Skin side |  | 31.89/no ct | no ct/36.73 | 36.59/37.37 | no ct/36.96 | no ct/no ct | no ct/no ct |
|  | Fur side | UV | 17.22/17.08 | 17.06/16.99 | 15.77/20.73 | 32.08/32.78 | 32.18/32.29 | 32.00/34.43 |
|  | Skin side |  | no ct/no ct | 37.21/no ct | no ct/no ct | no ct/37.69 | no ct/no ct | no ct/no ct |
|  | Fur side | Heat | 17.61/17.10 | 16.84/16.99 | 30.82/16.64 | 30.83/33.25 | 31.57/32.17 | 34.99/35.29 |
|  | Skin side |  | no ct/no ct | no ct/no ct | no ct/no ct | no ct/no ct | no ct/no ct | 38.74/38.18 |
| Fake fur | Fur side | None | 17.75/17.78 | 17.41/17.40 | 31.06/29.76 | 31.91/33.77 | 34.89/35.18 | no ct/no ct |
|  | Skin side |  | 17.33/17.04 | 16.55/17.07 | 35.84/36.67 | 35.88/no ct | no ct/37.57 | no ct/38.98 |
|  | Fur side | UV | 31.86/32.23 | 31.94/16.56 | 33.36/31.14 | 33.85/36.20 | 35.78/37.88 | no ct/no ct |
|  | Skin side |  | 33.85/33.65 | 17.34/17.33 | 35.82/37.69 | no ct/37.89 | no ct/38.78 | no ct/no ct |
|  | Fur side | Heat | 32.85/33.47 | 30.95/30.85 | 32.10/32.45 | no ct/no ct | 38.80/no ct | no ct/no ct |
|  | Skin side |  | 34.12/35.10 | 32.77/31.95 | no ct/no ct | no ct/no ct | no ct/no ct | no ct/no ct |
| Ctrl | Petri dish | None | 24.91/17.78 | 15.55/16.16 | 17.12/18.51 | 30.80/17.04 | 37.71/33.44 | 31.70/31.37 |
|  |  | UV | 30.45/30.64 | 31.61/31.64 | 29.62/30.11 | 30.08/28.74 | 32.08/31.75 | 30.00/29.24 |
|  |  | Heat | 29.59/28.88 | 31.98/28.78 | 29.38/29.93 | 30.84/28.97 | 31.59/30.00 | 30.92/30.94 |

Table S2: PCR results from the culture media of the test about the stability of SARS-CoV-2 on surfaces without inactivating treatment and after inactivating treatments in 60 ᵒC for 1 h. Ct-threshold has been set to 0.02.

| Surface | Treatment | 0 days | 1 day | 2 days | 5 days | 6 days | 7 days | 10 days |
| --- | --- | --- | --- | --- | --- | --- | --- | --- |
| Mink pelt, fur side | None | 17.28/17.00 | 18.58/18.26 | 20.84/18.47 | 18.65/18.81 | 17.90/18.60 | 18.75/17.73 | 17.99/19.26 |
|  | Heat |  |  |  |  |  | 35.52/35.87 |  |
| Mink pelt, skin side | None | no ct/18.21 | no ct/35.96 | no ct/39.19 | 37.63/36.47 | no ct/no ct | 37.89/no ct | no ct/38.87 |
|  | Heat |  |  |  |  |  | no ct/no ct |  |
| Fake fur, furside | None | 16.80/16.67 | 31.79/31.57 | 30.67/30.36 | 33.39/31.92 | 32.31/30.52 | 38.94/38.49 | no ct/38.00 |
|  | Heat |  |  |  |  |  | no ct/no ct |  |
| Fake fur, fabric side | None | 17.12/17.24 | 19.11/21.90 | 39.48/39.98 | 34.29/34.60 | 38.62/37.67 | 38.87/37.58 | no ct/36.70 |
|  | Heat |  |  |  |  |  | no ct/no ct |  |
| Leather | None | 17.68/16.98 | 33.53/33.98 | 34.80/35.62 | 37.95/37.87 | 36.28/30.67 | 38.98/35.48 | 38.45/35.15 |
|  | Heat |  |  |  |  |  | 37.55/no ct |  |
| Cotton | None | 37.81/38.13 | no ct/no ct | 37.07/no ct | no ct/no ct | no ct/no ct | no ct/no ct | no ct/no ct |
|  | Heat |  |  |  |  |  | no ct/no ct |  |
| Synthetic fabric | None | 38.15/18.01 | no ct/no ct | 38.38/no ct | 38.74/no ct | no ct/no ct | no ct/no ct | no ct/no ct |
|  | Heat |  |  |  |  |  | no ct/no ct |  |
| Petri dish | None | 16.18/16.98 | 18.16/18.11 | 18.50/18.72 | 19.33/23.07 | 33.32/30.06 | 29.05/33.82 | 33.74/32.39 |
|  | Heat |  |  |  |  |  | 33.82/34.91 |  |
| pos ctrl |  |  |  |  |  |  | 18.25/18.32 |  |
| neg ctrl |  |  |  |  |  |  | no ct/no ct |  |
